# Profiling the Blood Compartment of Hematopoietic Stem Cell Transplant Patients During Human Cytomegalovirus Reactivation

**DOI:** 10.3389/fcimb.2020.607470

**Published:** 2021-01-08

**Authors:** Biana Bernshtein, Aharon Nachshon, Miri Shnayder, Lauren Stern, Selmir Avdic, Emily Blyth, David Gottlieb, Allison Abendroth, Barry Slobedman, Noam Stern-Ginossar, Michal Schwartz

**Affiliations:** ^1^ Department of Molecular Genetics, Weizmann Institute of Science, Rehovot, Israel; ^2^ Discipline of Infectious Diseases and Immunology, Faculty of Medicine and Health, Charles Perkins Centre, University of Sydney, Camperdown, NSW, Australia; ^3^ Sydney Cellular Therapies Laboratory, Westmead Institute for Medical Research, Westmead, NSW, Australia; ^4^ BMT and Cell Therapies Unit, Westmead Hospital, Sydney, NSW, Australia; ^5^ Westmead Institute for Medical Research at the University of Sydney, Westmead, NSW, Australia; ^6^ Sydney Medical School, Faculty of Medicine and Health, University of Sydney, Camperdown, NSW, Australia

**Keywords:** human cytomegalovirus, blood compartment, hematopoietic stem cell transplantation, reactivation, peripheral blood mononuclear cell

## Abstract

Human cytomegalovirus (HCMV) is a widespread pathogen establishing a latent infection in its host. HCMV reactivation is a major health burden in immunocompromised individuals, and is a major cause of morbidity and mortality following hematopoietic stem cell transplantation (HSCT). Here we determined HCMV genomic levels using droplet digital PCR in different peripheral blood mononuclear cell (PBMC) populations in HCMV reactivating HSCT patients. This high sensitivity approach revealed that all PBMC populations harbored extremely low levels of viral DNA at the peak of HCMV DNAemia. Transcriptomic analysis of PBMCs from high-DNAemia samples revealed elevated expression of genes typical of HCMV specific T cells, while regulatory T cell enhancers as well as additional genes related to immune response were downregulated. Viral transcript levels in these samples were extremely low, but remarkably, the detected transcripts were mainly immediate early viral genes. Overall, our data indicate that HCMV DNAemia is associated with distinct signatures of immune response in the blood compartment, however it is not necessarily accompanied by substantial infection of PBMCs and the residual infected PBMCs are not productively infected.

## Introduction

Human cytomegalovirus (HCMV) is a widespread pathogen infecting most of the population worldwide. Like other herpesviruses, following primary infection, HCMV establishes a latent infection that persists for the lifetime of the host. Although HCMV infection in healthy individuals is mostly asymptomatic, reactivation from latency in immunocompromised individuals constitutes a serious health burden. For hematopoietic stem cell transplantation (HSCT) patients HCMV reactivation is a major risk factor (Stern et al., 2019). Reactivation in these patients can lead to HCMV disease that manifests in diverse symptoms, from gastroenteritis to respiratory symptoms, hepatitis and retinitis, and is also associated with graft versus host disease (Cantoni et al., 2010; Ljungman et al., 2017). Overall, HCMV reactivation is a leading infectious cause of morbidity and mortality in HSCT patients (Broers et al., 2000). Since HCMV reactivation is associated with DNAemia, i.e. the detection of viral DNA in the blood, HSCT patients undergo routine surveillance of HCMV DNA levels in the blood during the post-transplant period; most commonly by qPCR analysis of blood samples (Emery et al., 2000). Pre-transplant HCMV serostatus of the donor and recipient is the major risk factor for HCMV reactivation and disease following HSCT, with HCMV seropositivity of the recipient conferring the highest risk (George et al., 2010; Webb et al., 2018). HCMV reactivation develops in more than 50% of the cases where the recipient was HCMV seropositive and the donor was seronegative (R+/D-), while in cases where the recipient was HCMV seronegative and the donor was HCMV seropositive (R-/D+) there is a ~10% risk.

Hematopoietic progenitor cells and monocytes are considered major reservoirs of HCMV latency in humans (Hahn et al., 1998; Slobedman and Mocarski, 1999; Goodrum, 2016) however, the specific source of HCMV reactivation in these patients remains elusive. Understanding the blood cell subsets that are infected with HCMV during reactivation, as well as the effect of HCMV reactivation on the blood compartment, is important in order to elucidate the role of the blood compartment in progression and control of infection and in dissemination. Previous analyses of viral load in primary blood mononuclear cells (PBMCs) relied on relative measurements using quantitative PCR or *in-situ* hybridization and provided a wide range of results (Saltzman et al., 1988; Boivin et al., 1999; Hassan-Walker et al., 2001), and detailed transcriptomic analyses were not performed in such samples.

In order to systematically and accurately characterize the infection of the blood compartment during HCMV reactivation in HSCT recipients, we analyzed PBMCs from HSCT patients that exhibited HCMV DNAemia. We used digital droplet PCR (ddPCR), which allows specific and highly sensitive absolute quantification of DNA even at low amounts, to determine infection of specific cell types in blood samples from patients exhibiting HCMV DNAemia. RNA sequencing was further applied to study the host transcriptome in PBMCs as well as to characterize the viral expression pattern in PBMCs from HCMV reactivating HSCT patients. We found that although HCMV DNA was detected in the plasma at high levels, PBMCs harbored extremely low levels of viral DNA, with monocytes generally exhibiting the highest viral loads. Analysis of the host transcriptome suggested the development of HCMV-specific T-cells and the involvement of regulatory T cells (Tregs) and additional immune pathways during HCMV DNAemia. Interestingly, viral transcript levels were very low, in line with low viral loads found in the different PBMC subsets, however the gene expression pattern that was detected resembled that of early stages of productive infection, indicating that these cells do not go through a full productive cycle. Taken together, our findings indicate that DNAemia in HCMV reactivating HSCT patients is not necessarily accompanied by substantial infection of PBMCs, but is nevertheless associated with evident immune response signatures.

## Materials and Methods

### Cells and Virus Stocks

Peripheral Blood Monouclear Cells (PBMC) were isolated from fresh venous blood, obtained from healthy donors, using Lymphoprep (Stemcell Technologies) density gradient. The cells were cultured in RPMI media (Beit-Haemek, Israel) supplemented with 10% fetal bovine serum (FBS), 2 mM L-glutamine and 100 units/ml penicillin and streptomycin (Beit-Haemek, Israel) at 37°C in 5% CO2. Primary human foreskin fibroblasts (ATCC CRL-1634) were maintained in DMEM with 10% fetal bovine serum (FBS), 2 mM L-glutamine, and 100 units/ml penicillin and streptomycin (Beit-Haemek, Israel).

The TB40/E virus containing an SV40-GFP tag (TB40/E-GFP) was described previously (Sinzger et al., 2008; O’Connor and Murphy, 2012). Virus was propagated by electroporation of infectious bacterial artificial chromosome (BAC) DNA into fibroblasts using the Amaxa P2 4D-Nucleofector kit (Lonza) according to the manufacturer’s instructions. Viral stocks were concentrated by centrifugation at 26000xg, 4°C for 120 min. Infectious virus yields were assayed on THP-1 cells (ATCC TIB-202).

### Infection Procedures

For experimental infection, PBMCs were infected at a multiplicity of infection (MOI) of 5 and fibroblasts were infected at an MOI of 1. Infection was carried out by incubation with the virus for 2 h followed by two washes to clear out viral particles.

### Cell Staining for Flow Cytometry and Sorting

Cells were counted, and stained in cold MACS buffer (PBS, 5% BSA, 2 mM EDTA). Cell staining was done using the following antibodies:

Anti-human-CD45 (Clone: HI-30, Biolegend), anti-human-HLA-DR, DP, DQ (clone: REA332, Miltenyi Biotec), anti-human-CD14 (Clone: M5E2, Biolegend), anti-human-CD16 (Clone:3G8, Biolegend), anti-human-CD19 (Clone: SJ25C1, Biolegend), anti-human-CD3 (Clone: OKT3, Biolegend), according to manufacturer’s instructions. Cells were analyzed and sorted on a BD FACSAriaIII.

### Detection of Viral Genomes by Digital PCR

Detection of viral DNA was done using the QX200 droplet digital PCR system (Bio-Rad), using FAM labeled HCMV primer and probe (Human CMV HHV5 kit for qPCR using a glycoprotein B target (PrimerDesign) and HEX labeled RPP30 copy number assay for ddPCR (Bio-Rad), as previously described (Shnayder et al., 2020). Cells were counted, dry pelleted, and stored at −80°C prior to DNA extraction. DNA was extracted from the cell pellet in a 1:1 mixture of PCR solutions A (100 mM KCl, 10 mM Tris–HCl pH 8.3, and 2.5 mM MgCl2) and B (10 mM Tris–HCl pH 8.3, 2.5 mM MgCl2, 0.25% Tween 20, 0.25% Non-idet P-40, and 0.4 mg/ml Proteinase K), for 60 min at 60°C followed by a 10 min 95°C incubation, according to the description in (Roback et al., 2001). To avoid biases due to small cell numbers, samples with cell number <1,500 were excluded.

### RNA Library Construction

RNA libraries were generated from samples of ~100,000 cells according to the MARS-seq protocol (Jaitin et al., 2014; Keren-Shaul et al., 2019).

### Sequencing and Data Analysis

RNA-Seq libraries (pooled at equimolar concentration) were sequenced using NextSeq 500 (Illumina), with read parameters: Read1: 72 cycles and Read2: 15 cycles.

Analysis of bulk MARS-seq was done as described previously (Shnayder et al., 2018). The number of Unique Molecular Identifiers (UMIs) were:

**Table:** 

	Low DNAemia	High DNAemia
patient 6	1890938	5301580
patient 7	1324199	2925446
patient 8	4780993	5068593

### Correlation Analysis

Pearson correlation between viral gene expression profiles was calculated using Morpheus (https://software.broadinstitute.org/morpheus/).

### Differential Expression and Enrichment Analysis

The differential expression analysis was done with DESeq2 (version 1.22.2) (Love et al., 2014) using default parameters, with the number of reads in each of the samples as an input.

## Results

### PBMC From Healthy Individuals Harbor HCMV Genomes Following Experimental Infection

**Figure 1:**
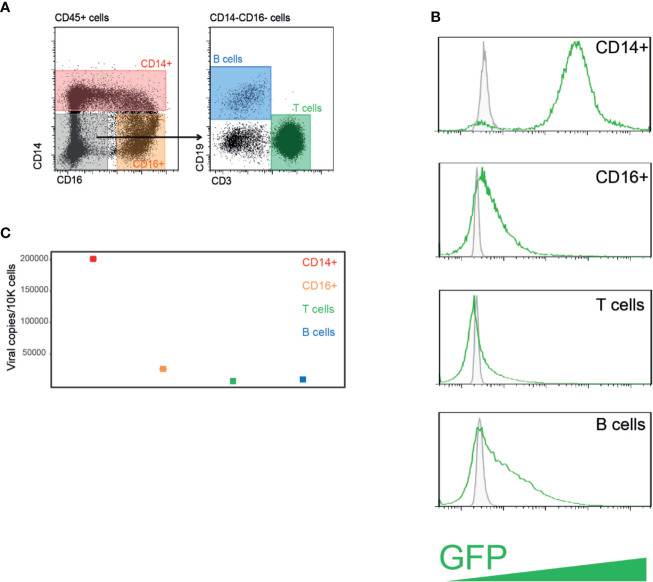
PBMCs from healthy individuals are efficiently infected with HCMV following experimental infection. **(A)** HCMV infected PBMCs were FACS sorted to four distinct cell populations. **(B)** Flow cytometry analysis of GFP expression levels in PBMCs experimentally infected with HCMV strain TB40/E-GFP at 22 h post infection. Uninfected control cells and infected cells are shown in grey and green lines, respectively. **(C)** Quantification of viral genomes by ddPCR in indicated PBMCs populations presented as copies per 10,000 cells. Graph reflects mean and 95% CV of Poisson distribution, calculated from two technical replicates per sample.

Following HSCT, patients are monitored for HCMV reactivation by means of measuring viral DNA loads either in whole blood or in plasma, however it is unclear what is the source of the detected viral DNA and which cells in the blood carry HCMV. To unbiasedly assess the ability of PBMCs to be efficiently infected with HCMV, we first purified PBMCs from the blood of a healthy donor and infected them at high MOI with an HCMV strain TB40/E-GFP, which expresses GFP from an SV40 promoter. Twenty-four hours post infection, cells were FACS sorted based on standard cell markers to distinct blood cell types: CD14+CD16- cells which are mainly classical monocytes, CD16+CD14- cells which include non-classical monocytes (Guilliams et al., 2018), a subset of NK cells (Lanier et al., 1989), and dendritic cells (Fromm et al., 2020), and T and B cells according to cell surface markers CD3 and CD19, respectively (**Figure 1A**). The remaining CD14-CD16-CD3-CD19- cells that were not sorted are most likely subsets of NK cells and dendritic cells. Flow cytometry analysis revealed that HCMV infects all cell types, as evident by appearance of GFP positive cells; however, CD14+ monocytes exhibited significantly higher percentage of GFP positive cells as well as much higher GFP intensity (**Figure 1B**), suggesting that they are most efficiently infected. It is noteworthy that none of these cell types are considered to support productive infection, and specifically monocytes, despite having a clear GFP signal, are latently infected (Shnayder et al., 2018).

Droplet digital PCR (ddPCR) is a relatively recent technology in which PCR amplification of a specific amplicon is partitioned into a large number of discrete reactions, allowing greater precision and reproducibility compared to conventional PCR based methodologies, and highly sensitive absolute quantification of nucleic acids (Hindson et al., 2013; Taylor et al., 2017). We used ddPCR to directly assess the viral load in these different cell populations. In agreement with GFP levels, all cell types harbored some HCMV genomes, while CD14+ monocytes exhibited the highest levels of HCMV genomes per cell (**Figure 1C**), indicating that CD14+ monocytes are much more permissive to HCMV experimental infection compared to the other blood cell types tested, which exhibited low level of infection.

### Low Levels of Viral DNA in Peripheral Blood Mononuclear Cells During Human Cytomegalovirus DNaemia

**Figure 2:**
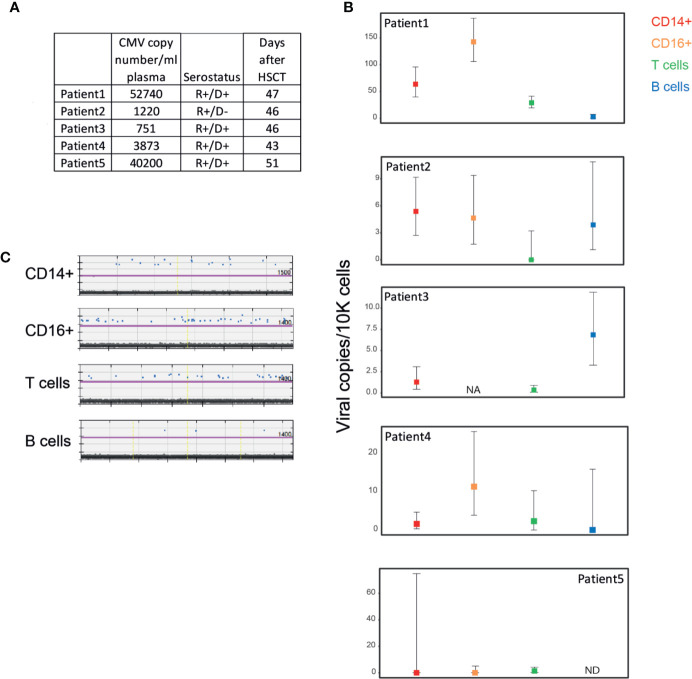
Low levels of HCMV DNA detected in PBMCs from HCMV reactivating HSCT patients during HCMV DNAemia. **(A)** Description of samples from five HCMV reactivating HSCT patients. HCMV copy number was measured by RT-qPCR. PBMCs from HSCT patients were sorted to four distinct cell populations as described in **Figure 1A**. **(B)** Quantification of viral genomes in the indicated PBMC populations from individual HSCT patients, presented as copies per 10,000 cells. Graph reflects mean and 95% CV of Poisson distribution, calculated from at least 2 technical replicates per sample. NA, not available; ND, not detected; i.e. positive event number in the sample is <=2. **(C)** ddPCR results of PBMC populations from patient 1, technical replicates separated by yellow vertical lines. The magenta line marks the threshold.

To define HCMV infection of different cell types in the blood during DNAemia, we analyzed PBMCs from HSCT recipients that exhibited HCMV reactivation, as defined by detectable levels of HCMV genomic DNA in the blood. These patients were periodically monitored for HCMV DNAemia following HSCT and none exhibited HCMV organ disease. PBMCs were purified from blood samples of five HSCT recipients taken at the peak of DNAemia, as measured by HCMV DNA levels in the plasma (**Figure 2A**). PBMCs were sorted to distinct cell populations as described in **Figure 1A** and viral load in these cells was measured by ddPCR. We set a cut-off of at least two positive events, which was determined according to analysis of samples from healthy sero-negative donors (**Supplementary Figure 1**). PBMCs from four of the patients exhibited extremely low (<15 genomes/10,000 cells) to undetectable HCMV genomic levels in all cell types, indicating an extremely low level of infected PBMCs in their blood (**Figure 2B**, patients 2–5). In one DNAemic sample (patient 1) there were relatively higher levels of HCMV genomic DNA, yet still very low, that reached ~50 genomes/10,000 CD14+ monocytes, ~150 genomes/10,000 CD16+ cells, ~35 genomes/10,000 T cells and undetected levels in B cells (**Figures 2B, C** **)**. Although higher viral loads were detected in this patient compared to the other four patients, and there was a preference towards the infection of CD16+ cells and CD14+ which include most of the monocytes, the viral loads were still very low and far from the viral load that could be achieved in experimental infection (**Figure 1C**). These results suggest that although PBMCs are permissive to experimental infection, infection levels are extremely low in the context of reactivation *in-vivo* following HSCT, and cannot explain the high HCMV DNA levels detected in the plasma.

### Transcriptional Changes During Human Cytomegalovirus DNAemia

**Figure 3:**
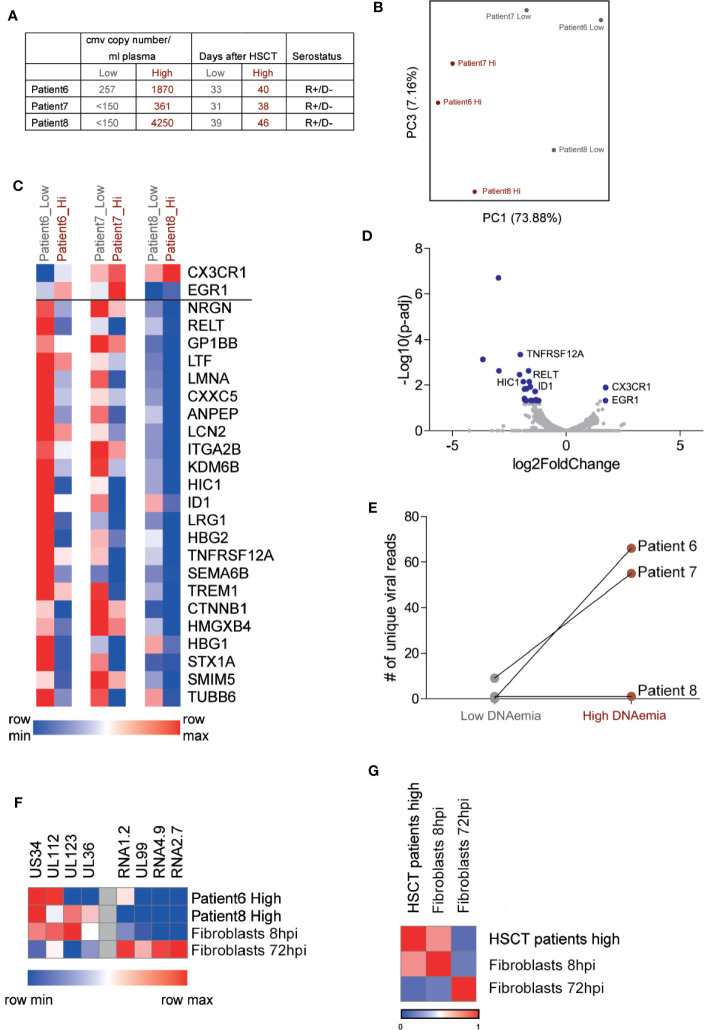
RNA sequencing analysis of PBMCs from HCMV reactivating HSCT patients reveals low viral transcript levels and a discernible host response to active HCMV infection. **(A)** Description of samples from three HCMV reactivating HSCT patients. HCMV copy number was measured by RT-qPCR. **(B)** Principal component analysis of the transcriptional profile of PBMCs from three HSCT patients at two time points. **(C)** Heat map of 25 host genes significantly differentially expressed (fold change>2 and FDR<0.05) in all three HSCT patients comparing samples of low and high DNAemia. **(D)** Volcano plot of statistical significance (-log10 p-value) against log 2 ratio of host transcript levels between low and high DNAemic samples of PBMCs from three HCMV reactivating HSCT patients, based on RNA-seq data. Blue dots mark significantly up or down regulated genes (fold change>2, adj-p value<0.05). **(E)** Total number of viral reads in PBMCs from low and high DNAemic samples of three HCMV reactivating HSCT patients. **(F)** Heat map showing the expression level of representative immediate early stage and late stage viral genes in PBMCs of HSCT patients or experimental lytically infected fibroblasts at 8 or 72 h post infection. **(G)** Heat map showing Pearson’s correlation between viral gene expression program in PBMCs from HSCT patients and experimental lytically infected fibroblasts at 8 or 72 h post infection.

To characterize the changes in PBMCs from HCMV reactivating HSCT patients during DNAemia and to examine the viral gene expression profile, we analyzed the transcriptome of PBMCs from three patients following HSCT by RNA-seq, at two time points: no detection or very low level of viral DNA in the blood and during measurable DNAemia (**Figure 3A**). These patients are at the stage of reconstitution of their immune system, which likely has a substantial impact on the transcriptional profile of their blood cells. Nevertheless, Principle Component Analysis (PCA) of RNA-seq data indicated that the high-DNAemic samples clustered separately from low-DNAemic samples of the same patient (**Figure 3B**).

Differential expression analysis revealed differences between high-DNAemic and low-DNAemic PBMCs that were shared between all three patients. 25 genes were significantly differentially expressed between the two sample types (adjusted p-value<0.05, fold change>2, **Figures 3C, D**, **Table S1**); two were upregulated and 23 were downregulated in high-DNAemic samples. The two upregulated genes in high-DNAemia were CX3CR1 and EGR1, both of which are expressed by HCMV specific T cells following HSCT (Hertoghs et al., 2010; Hardy et al., 2018). Among downregulated genes were several genes related to inflammatory responses, including two genes belonging to the TNF-receptor superfamily (RELT, TNFRSF12a), as well as genes related to innate immune responses (LCN2, ITGA2B). Interestingly, two downregulated genes, HIC1 and ID1, are known as transcription factors that enhance Treg function and differentiation (Liu et al., 2014; Ullah et al., 2018). Thus, we were able to readily detect a response of blood cells to HCMV DNAemia, which was captured by reproducible transcriptional changes. Although these genes are potentially related to HCMV reactivation, they may also be related to immune reconstitution or other processes in the immune system of these patients.

In line with the extremely low viral DNA level found in these cells, analysis of viral gene expression indicated very low to undetectable viral transcript levels even in the high DNAemic samples (**Figure 3E**, **Table S2**). In two high-DNAemia samples, we found 55 and 66 unique viral reads in ~ 5 million total reads, while in the third patient there were only two viral reads (**Figure 3E**, **Table S2**). Surprisingly, the viral transcripts detected in these samples were mainly of genes that are expressed at an immediate early time point such as UL123 and UL36, while the level of transcripts that are abundant at the late stages of productive infection was much lower (**Figure 3F**). Indeed, although the number of viral reads we obtained is low, comparison of viral gene expression pattern of these samples to the expression pattern of early and late stages of productive infection, based on RNA-seq analysis of infected fibroblasts at 8 and 72 hpi, revealed high correlation with early stage of infection (R=0.71, **Figure 3G**). The absence of late transcripts indicates that the cells from which these viral transcripts originate are not productively infected and thus are unlikely to produce infectious virus. Overall, the transcriptome analysis of PBMCs from HSCT patients reveals host response to HCMV infection and low viral gene expression, which resembles early stages of productive infection without clear evidence of a full replicative cycle.

## Discussion

HCMV reactivation constitutes a major clinical burden following bone-marrow as well as solid organ transplantation. Reactivation is detected in patients by measurement of HCMV DNA in the blood. Despite the importance of understanding the role of the blood compartment in HCMV infection and dissemination following reactivation of the virus, the nature of this infection is not well characterized. Depicting the blood cell types that are infected, portraying the nature of viral infection in these cells and its impact on the host will provide insight on these issues.

Quantitative assessments of viral load during HCMV reactivation in different blood cell types were done in the past but relied on less quantitative methodologies than the ones currently available (Saltzman et al., 1988; Boivin et al., 1999; Hassan-Walker et al., 2001). ddPCR is a relatively recent technique allowing absolute measurements of nucleic acids with superb precision and reproducibility (Hindson et al., 2013; Taylor et al., 2017). To delineate the levels of HCMV infection in different PBMC populations, we used this highly sensitive tool to measure the absolute level of HCMV genomes in the different cell types. We show that CD14+ monocytes are markedly the preferential target of HCMV infection following experimental infection of PBMCs. CD14+ monocytes are considered sites of HCMV latency, which may reactivate in response to differentiation (Goodrum, 2016). These cells were indeed very efficiently infected as apparent from the level of the GFP reporter as well as from the amount of viral genomes that were detected in these cells, however this infection is not productive as viral gene expression is repressed and infectious virus is not produced (Shnayder et al., 2018). In comparison, CD16+ cells, B cells, and T cells, are much less efficiently infected. This is in line with the prevalent view of monocytes as the main cell type in the blood to be infected by HCMV.

ddPCR measurements in samples from HSCT recipients with HCMV reactivation, at the peak of DNAemia, also supported the notion that monocytes are generally the most efficiently infected cell type among PBMCs, although as CD16+ cells showed higher viral DNA levels in some of the patients, this may implicate additional cell types. However the levels of infection in all blood cell types tested are extremely low in these settings. This suggests that much of the DNA measured in the plasma does not originate from mononuclear cells in the blood, and perhaps does not reflect infectious virus in the blood. The difference in viral load between experimental infection and in the context of reactivation *in-vivo* after HSCT probably stems from several factors that greatly differ between natural and experimental systems including the high MOI that is used in experimental settings, the viral strain and changes in the environment of the cells.

In light of previous studies, showing much higher levels of HCMV DNA in mononuclear blood cells, the extremely low infection levels we find are surprising. This difference may represent variability between patients or may be related to the more precise measurement method. It is noteworthy that our analysis includes patients with very high DNAemia, negating the possibility that we screened only patients with low levels of HCMV reactivation. We cannot rule out the possibility that in other patients there may be higher levels of HCMV genomes in PBMCs during DNAemia, however in a previous study we found extremely low levels of HCMV genomes in monocytes of additional HCMV reactivating HSCT patients (Shnayder et al., 2020). Further research will be required to delineate the source of HCMV genomes in the blood of patients with low PBMC infection during DNAemia. A possible target is polymorphonuclear cells which were shown to be infected during HCMV reactivation (Saltzman et al., 1988; Hassan-Walker et al., 2001). Interestingly, circulating cytomegalic endothelial cells have been identified in the blood of solid organ transplant patients, AIDS patients, and HSCT patients (Salzberger et al.; Grefte et al., 1993; Percivalle et al., 1993; Gerna et al., 1998).

Analysis of viral transcripts in PBMCs from DNAemic samples supports the notion that these cells are hardly infected with HCMV, as the levels of viral mRNAs were extremely low. Intriguingly, the dominant viral genes that were expressed were immediate early (IE) genes. We previously examined HCMV gene expression in diverse human tissues by analyzing RNA-seq samples from the Genotype-Tissue Expression (GTEx) Consortium. Interestingly, this analysis also uncovered samples with a restrictive gene expression pattern that includes mainly IE transcripts and these were specifically found in blood samples (Shnayder et al., 2018). This appearance of the same pattern implies that there are blood cells with limited viral gene expression that might reflect a threshold that needs to be crossed before the virus can accomplish the complete infection cycle. This specific pattern also suggests that the PBMCs that are infected in these DNAemic samples are not productively infected.

The viral genome levels we find in PBMCs from DNAemic samples are very similar to the levels that were estimated for PBMCs during latency (Slobedman and Mocarski, 1999; Jackson et al., 2017), which may suggest that these could be latent cells which are not related to reactivation. However, several lines of evidence suggest that they are related to reactivation. First, some of the samples used in the study are from R+/D- cases, where latent blood cells are not expected. Second, although the transcript levels are extremely low, the transcription profile is very distinct from what was described for latent monocytes (Cheng et al., 2017; Shnayder et al., 2018). Third, beside patient 5, there is an association between the levels of DNAemia in the patient and the levels of viral genome copies that were measures in PBMCs by ddPCR. Thus although the infection is low, we suspect that it is related to the reactivation of HCMV.

Despite the extreme changes in the blood compartment during reconstitution of the immune system following HSCT, the gene expression profile of high-DNAemic samples clustered away from low-DNAemic samples from the same patient and there were reproducible transcriptional changes. This suggests that these genes are associated with HCMV reactivation although it possible that they are related to other immune processes, e.g. immune reconstitution. HCMV is known to elicit a robust CD8+ T cell response. One of the upregulated genes in high-DNAemic samples, CX3CR1, is upregulated in HCMV specific T-cells following HCMV infection (Hertoghs et al., 2010). The expression of EGR1, which was also upregulated in our data, was characteristic of CMV-specific T-cells in non-immune reactive HSCT patients, which are associated with poor CMV control (Hardy et al., 2018). These results suggest that an immune cellular response to HCMV reactivation has developed in these patients. Regulatory T cells (Tregs) are essential for regulating the function of effector T cells. The proportion of Tregs within CD4+ T-cell population was found to decrease during HCMV reactivation in HSCT patients (Velaga et al., 2013). Notably, two out of the 23 down-regulated genes in samples with HCMV DNAemia, Hic1 and ID1, were shown to promote Treg differentiation, expansion and suppression functions, supporting decrease of Treg functions during HCMV reactivation (Liu et al., 2014; Ullah et al., 2018). In addition, several genes related to TNF signaling and NF-kappa-b activation, or associated with innate immune response, as well as antiviral processes were also downregulated. Further studies will need to establish the importance and function of these changes during HCMV reactivation in HSCT patients.

Overall, our data suggest that DNAemia in HCMV reactivating HSCT patients is not necessarily accompanied by substantial infection of PBMCs, and that the infected PBMCs are not productively infected. Nevertheless, high DNAemia in these patients is associated with transcriptional changes that indicate an active immune response. Our findings elucidate the nature of HCMV infection in PBMCs during HCMV reactivation in HSCT patients and shed light on the role of the blood compartment in progression and control of HCMV infection.

## Data Availability Statement

All next-generation sequencing data files were deposited in Gene Expression Omnibus under accession number GSE161752.

## Ethics Statement

The studies involving human participants were reviewed and approved by the Weizmann Institutional Review Board (IRB application 92–1) and Human Research Ethics Committee of the University of Sydney and the Western Sydney Local Health District. Informed consent was obtained from all study participants prior to enrollment in accordance with the Declaration of Helsinki. The patients/participants provided their written informed consent to participate in this study.

## Author Contributions 

BB, BS, NS-G, and MSc designed the research. BB and MSh performed the research. LS, SA, EB, DG, AA, and BS provided critical reagents and advice. BB, AN, NS-G, and MSc analyzed the data, and BB and MSc wrote the paper. All authors contributed to the article and approved the submitted version.

## Funding

This study was supported by ERC-CoG-2019- 864012.

## Conflict of Interest

The authors declare that the research was conducted in the absence of any commercial or financial relationships that could be construed as a potential conflict of interest.

## Supplementary Material

Supplementary Figure 1 ddPCR Analysis of Samples From Healthy Sero-Negative Donors. ddPCR results of PBMC populations from two healthy HCMV sero-negative donors, technical replicates separated by yellow vertical line. The magenta line marks the threshold.
