# Supplementary figures and images for: Profiling the Blood Compartment of Hematopoietic Stem Cell Transplant Patients During Human Cytomegalovirus Reactivation
Source: Front Cell Infect Microbiol. 2021 Jan 8;10:607470. doi: 10.3389/fcimb.2020.607470 (PMC7820775; doi:10.3389/fcimb.2020.607470)

# Supplementary Figure 1

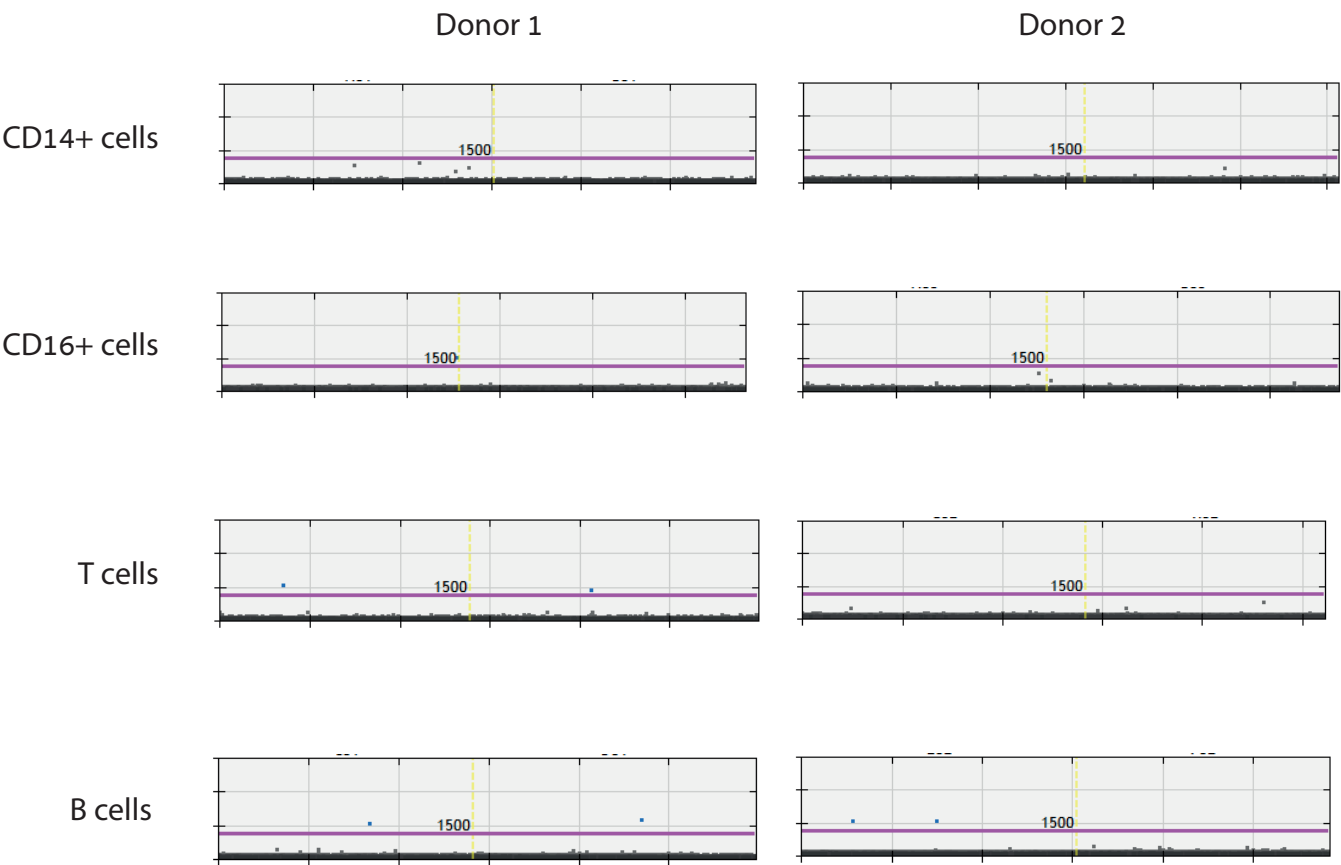

Supplement: Supplementary Figure 1 — ddPCR Analysis of Samples From Healthy Sero-Negative Donors. ddPCR results of PBMC populations from two healthy HCMV sero-negative donors, technical replicates separated by yellow vertical line. The magenta line marks the threshold. [file DataSheet_1.pdf]
